# Old Bug, New Challenge: Invasive Group A Streptococcus and a Rare Pediatric Holospinal Epidural Abscess

**DOI:** 10.7759/cureus.98990

**Published:** 2025-12-11

**Authors:** Natalia Syrimi, Despoina Maritsi, Alma Sulaj, Maria Tsolia, Angeliki Syngelou

**Affiliations:** 1 Second Department of Paediatrics, Panagiotis and Aglaia Kyriakou Children's Hospital, National and Kapodistrian University of Athens, Athens, GRC

**Keywords:** holospinal abscess, holospinal epidural abscess, igas, invasive group a streptococcus, pediatrics, spinal epidural abscess

## Abstract

Invasive group A Streptococcus (iGAS) infections have risen in the post-COVID-19 era, with significant morbidity and mortality in children. Spinal epidural abscess (SEA) is a rare, life-threatening condition, and pediatric cases are scarcely reported. SEA caused by iGAS is even more unusual, with only sporadic case reports in the literature. Herein, a challenging case of pediatric SEA due to iGAS is described, raising awareness of this clinical entity.

An otherwise healthy 18-month-old girl presented with a three-day history of gait disturbance, high temperature, and seizures. Lumbar puncture revealed pus, and cultures grew group A Streptococcus. Magnetic resonance imaging demonstrated a holospinal epidural abscess. The patient was managed conservatively with serial epidural needle drainage and prolonged intravenous antibiotics. She achieved complete clinical and radiological recovery without neurological sequelae.

Pediatric SEA is rare, but early recognition and appropriate management are essential to prevent irreversible complications. Clinicians should remain vigilant for spinal complications of iGAS infections to optimize outcomes.

## Introduction

Since the lifting of non-pharmaceutical interventions (NPIs) against SARS-CoV-2 transmission, many European countries have reported a surge in invasive group A Streptococcus (iGAS) infections, with increased morbidity and mortality in children [1,2]. The spike observed in 2022-2023 has encompassed a wide range of clinical manifestations [3].

Spinal epidural abscess (SEA) in children is a rare invasive bacterial infection characterized by the accumulation of purulent material between the dura mater, the outermost membrane covering the spinal cord, and the spinal canal. It is a potentially life-threatening condition that also carries the risk of irreversible neurological damage and paralysis [4]. Diagnosis is often challenging, as the classic clinical triad of back pain, fever, and neurological deficit is not consistently present in pediatric patients [5].

The most common causative pathogen is Staphylococcus aureus (accounting for approximately 60-70% of cases), whereas Gram-negative organisms and streptococcal species, including group A Streptococcus, are less frequently identified. Predisposing factors include previous lumbar puncture, epidural anesthesia, spinal surgery, long-term corticosteroid use, and underlying systemic diseases such as sickle cell anemia, leukemia, or other immunodeficiencies [6]. Reports in the literature have also implicated prior varicella infection as a potential risk factor for pediatric iGAS-associated SEA [7,8].

The mid-thoracic to lumbar spine is the most common site of involvement, likely due to the relative narrowing of the spinal cord and the wider epidural space in these regions. The epidural venous plexus is considered the most probable route of infection. Most SEAs are dorsally located, while ventral abscesses are uncommon because the posterior longitudinal ligament adheres tightly to the ventral dura mater, leaving only a minimal potential space [6].

Available pediatric data are largely limited to case reports and small series, providing insufficient evidence to establish standardized management guidelines [4]. Treatment typically involves surgical decompression and drainage combined with systemic antibiotic therapy, although conservative, non-surgical management with prolonged antimicrobial therapy alone may be appropriate in selected cases. A non-operative approach can be considered in patients without neurological deficits, patients with extensive multilevel disease that would require a wide laminectomy, in cases of paraplegia exceeding 48 hours, or when multiple comorbidities elevate surgical risk [5].

Herein, we describe a pediatric case of extensive holospinal SEA secondary to iGAS, an unusually severe presentation that distinguishes this case from typical localized iGAS infections. This case was successfully managed with a minimally invasive drainage technique in combination with systemic antimicrobial therapy. We aim to raise clinical awareness of this rare but serious condition and discuss the diagnostic and therapeutic challenges encountered in managing such extensive iGAS-associated SEA.

## Case presentation

An 18-month-old previously healthy girl was admitted with a three-day history of unsteady gait, an initially subtle motor change that would prove to be an important early warning sign. One week earlier, she had mild gastroenteritis with low-grade fever and diarrhea, which resolved spontaneously.

On arrival at the emergency department, she was febrile and tachycardic and developed a generalized seizure, requiring admission to the pediatric intensive care unit (PICU). She was intubated and ventilated for several hours. Post-extubation, examination revealed pharyngeal erythema, neck stiffness, and meningeal irritation. She had no relevant medical or surgical history and was fully immunized according to the Greek National Vaccination Program.

Laboratory investigations showed leukocytosis (20,400/μL; 88% neutrophils) and elevated C-reactive protein (268 mg/L). Biochemistry, urinalysis, chest radiograph, and brain computed tomography (CT) scan were unremarkable. Lumbar puncture yielded thick purulent fluid with marked pleocytosis (500,000 cells/μL, 85% neutrophils, 10% lymphocytes), and Gram-positive cocci were observed in direct microscopy (Table 1).

**Table 1:** Summary of laboratory results. WBC: white blood cells; CRP: C-reactive protein; CSF: cerebrospinal fluid; CT: computed tomography.

Parameter	Result	Units	Reference range/normal values	Interpretation
Total leukocyte count (WBC)	20,400	/µL	4,000 – 10,000/µL	Markedly elevated (leukocytosis)
Neutrophils	88	%	40 – 75%	Neutrophilia
CRP	268	mg/L	<5 mg/L	Strongly elevated (Severe inflammation/infection)
Lumbar puncture appearance	Thick purulent fluid	—	Clear, colorless	Abnormal — suggests bacterial infection
CSF total cell count	500,000	cells/µL	<5 cells/µL	Marked pleocytosis
CSF neutrophils	85	%	0 – 6%	Predominant neutrophilic response
CSF lymphocytes	10	%	40 – 80%	Relative lymphopenia
Gram stain	Gram-positive cocci	—	None seen	Suggestive of bacterial meningitis (likely Streptococcus spp.)
Biochemistry	Unremarkable	—	Within normal limits	Normal
Urinalysis	Unremarkable	—	Within normal limits	Normal
Chest radiograph	Unremarkable	—	Clear lungs	Normal
Brain CT scan	Unremarkable	—	No acute pathology	Normal

**Figure 1:**
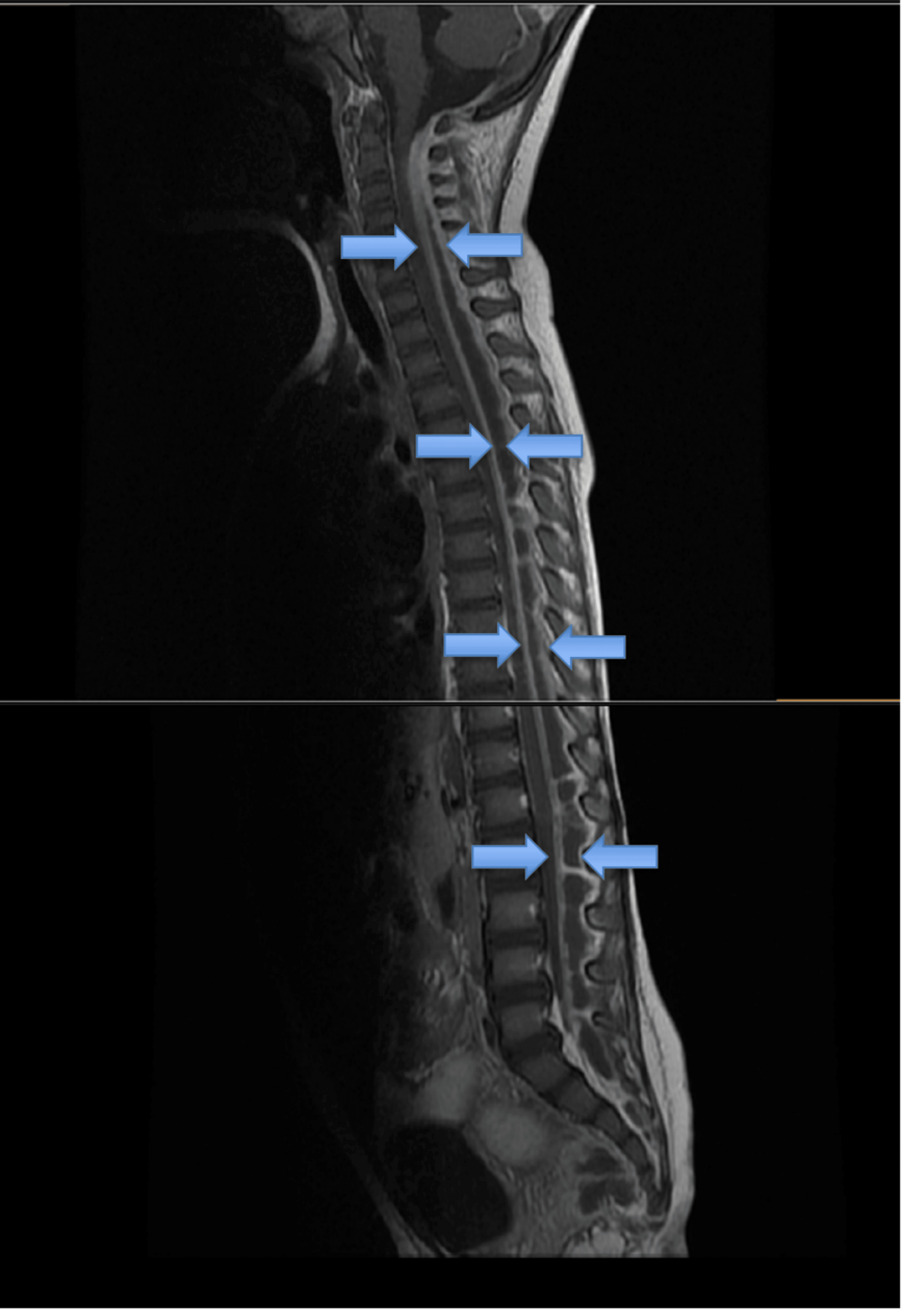
MRI at presentation (day one). Contrast-enhanced sagittal T1-weighted MRI depicting a holospinal epidural abscess extending from the cervical to the sacral spine, compressing the spinal cord.

Due to the inconsistency of laboratory findings and clinical presentation, where refusal to walk was the first and prominent symptom in the absence of fever, drowsiness, headache, and confusion, a strong suspicion of an alternative diagnosis other than bacterial meningitis was raised among clinicians. The prominence of motor symptoms with preserved mental status post extubation prompted an overall consensus to perform an urgent MRI scan of both brain and spine. This revealed a holospinal epidural abscess extending from the cervical to the sacral spine, without significant meningeal inflammation and without intracranial lesions. The spinal cord appeared compressed (Figure 1).

It was retrospectively understood that the lumbar puncture had accessed the epidural space rather than the subarachnoid area, and the sample represented epidural pus rather than cerebrospinal fluid, a finding that helped explain the discordance between laboratory results and clinical presentation.

Empirical ceftriaxone and vancomycin were started, later narrowed to high-dose intravenous ampicillin (300 mg/kg/day) and clindamycin (40 mg/kg/day) when cultures from blood, throat, and pus grew group A Streptococcus (emm type 12). Antibiotics were administered for a total duration of four weeks and seven days, respectively.

The patient became afebrile by day four, with normalization of inflammatory markers. A multidisciplinary team, including infectious disease specialists, pediatricians, neurosurgeons, and radiologists, opted for conservative management. Serial epidural needle drainage was performed, yielding 20 mL (day one), 3 mL (day two), and 20 mL (day four) of pus.

**Figure 2:**
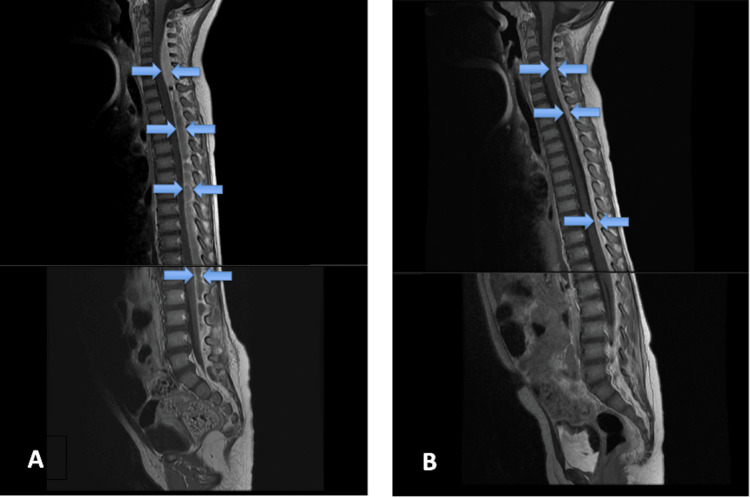
Follow-up contrast-enhanced, sagittal T1-weighted MRI views. (A) On day 10, there is a reduction in epidural collections at the thoracic, lumbar, and sacral levels, with a small residual collection at the cervical level. The dura mater appears thickened with evident contrast enhancement. (B) On day 15, there is further reduction of the cervical epidural pus, and the cord is nearly fully decompressed.

Follow-up MRI at day 10 showed near-complete resolution of thoracolumbar and sacral abscesses, with a small cervical residual. The child remained afebrile, and she was gradually able to ambulate. Further improvement of her clinical and radiological condition was noted on day 15 with normalization of inflammatory markers and further reduction of the cervical epidural pus (Figure 2).

**Figure 3:**
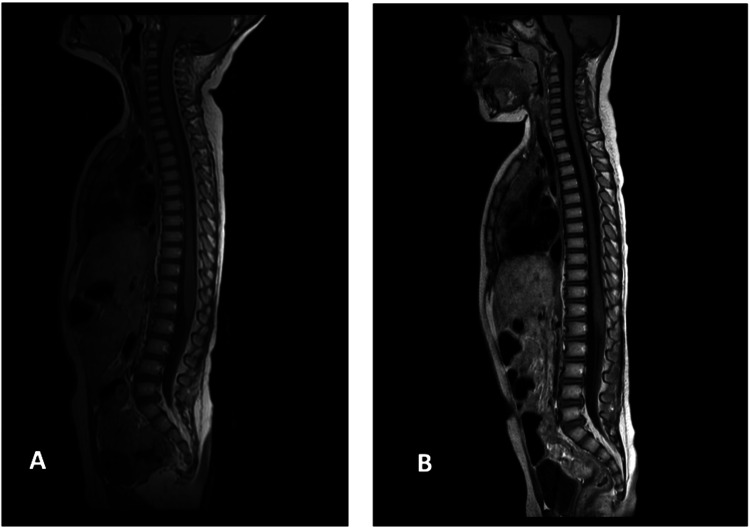
Later follow-up contrast-enhanced, sagittal T1-weighted MRI views. (A) On day 30, there is a complete radiological resolution. (B) One month post-discharge repeat MRI shows normal spinal appearance with no residual abscess or compression, confirming long-term resolution.

On day 30, MRI confirmed complete resolution of the lesions, and the girl was discharged home with no neurological or musculoskeletal deficits. At two months post discharge, she remained well and neurologically intact with excellent lower extremity strength and motor activity. Repeat MRI confirmed sustained resolution of the epidural abscess (Figure 3).

## Discussion

This is a case report of an extremely rare and severe iGAS infection presenting with a holospinal epidural abscess in a previously healthy toddler. The main challenges in this case were the absence of the classic clinical triad at initial presentation and the ambiguous, limited data on pediatric SEA, with no established consensus on optimal management. Although meningitis was initially suspected, as the child presented with high fever and seizures, and purulent fluid was obtained via lumbar puncture, she remained alert, active, and fully oriented, showing no signs of altered mental status suggestive of central nervous system infection. These findings prompted further evaluation with spinal MRI, which ultimately established the diagnosis of SEA. The most critical diagnostic clue was the child's initial presentation with refusal to walk and unsteady gait-motor changes that preceded systemic signs of infection. Recognition of these early, subtle motor signs in the context of systemic infection is essential for the timely diagnosis of pediatric epidural abscess.

iGAS infections have shown an increase in the post-COVID-19 era. Current evidence identifies several predisposing factors, including an “immune gap” attributed to pandemic-related restrictive measures, co-infection with respiratory viruses, and the emergence of new, more virulent strains, most notably the M1UK clade, which has become the dominant cause of invasive infections worldwide [9]. In our case, a preceding viral illness manifested as a self-limited mild gastroenteritis. The detected emm type 12 strain is among the leading emm types associated with iGAS reported in the literature [9]. Furthermore, only a handful of pediatric cases have been described in which iGAS was identified as the causative pathogen of spinal epidural abscess [4,8,10].

Although there is no consensus on the optimal management of SEA, the standard approach typically involves surgical decompression and drainage, combined with several weeks of systemic antibiotic therapy. Most available data are extrapolated from adult studies, with limited evidence in the pediatric population [9,11]. An institutional case review published by Hawkins et al. is the only recent pediatric report on the successful management of seven out of nine SEA cases with non-surgical management [12,13]. In our case, conservative treatment was deemed more appropriate, given the absence of neurological deficits at presentation and the extensive involvement of the entire spinal axis. Therefore, a minimally invasive approach using epidural needle drainage in conjunction with continuous antibiotic therapy was selected. This strategy avoided the risks associated with extensive spinal surgery while achieving adequate source control of infection. Informed consent was obtained from the parents after thorough discussion of both surgical and conservative management options, including the risks and benefits of the chosen unconventional approach, given the extent of the disease.

The patient underwent a total of four MRI scans (at presentation, day 10, day 15, and day 30) to monitor treatment response and ensure complete resolution of the abscess. All imaging studies were performed as part of routine clinical care and covered by the national healthcare system. Serial imaging was essential for guiding the duration of therapy and confirming the safety of the conservative approach. The favorable clinical outcome in this case supports the feasibility of minimally invasive management in selected pediatric patients with extensive SEA, even in the context of holospinal involvement, when close monitoring and appropriate antimicrobial therapy are employed.

## Conclusions

Holospinal epidural abscess in children caused by iGAS is an extremely rare and life-threatening condition, with limited data guiding prompt diagnosis and optimal management. Our experience demonstrates that the minimally invasive drainage technique combined with systemic antimicrobial therapy may be a prudent therapeutic option in carefully selected pediatric patients. As iGAS infections continue to rise globally, clinicians should maintain a high index of suspicion for this rare but life-threatening disease.
